# Clinical Review and Prognostic Analysis of α-Amino-3-Hydroxy-5-Methyl-4-Isoxazole Propionate Receptor-Associated Encephalitis

**DOI:** 10.3389/fneur.2021.665229

**Published:** 2021-05-13

**Authors:** Kemo Wang, Yanting Shi, Qianwen Du, Ran-ran Zhang, Huaikuan Wu, Shan Qiao, Xuewu Liu

**Affiliations:** ^1^Department of Neurology, Qilu Hospital, Cheeloo College of Medicine, Shandong University, Jinan, China; ^2^Department of Neurology, The First Affiliated Hospital of Shandong First Medical University, Jinan, China; ^3^Institute of Epilepsy, Shandong University, Jinan, China

**Keywords:** neuroimmune, amnesia, seizure, antibodies, immunotherapy, outcome

## Abstract

**Purpose:** Autoimmune encephalitis (AE) is a heterogeneous neurological autoimmune disorder associated with cognitive and psychiatric symptoms. It can be divided into several subtypes based on autoantibodies. Anti-α-amino-3-hydroxy-5-methyl-4-isoxazolepropionic acid receptor encephalitis (AMPAR-E) is one of the recently discovered AE subtypes, usually manifesting limbic encephalitis and with a good prognosis. Considering AMPAR-E has been described for the first time, only a few cases with similar antibodies have been reported clinically. We aimed to clarify the clinical course and prognosis of the disease in the light of previous reports.

**Patients and Methods:** We collected data on the diagnosis and treatment of six cases of AMPAR-E, diagnosed at the Qilu Hospital of Shandong University in the past 5 years. We retrospectively analyzed the clinical characteristics of the patients and performed a follow-up of the disease.

**Results:** The patients often presented with limbic encephalitis, which sometimes coexisted with tumors. In addition, immunotherapy had a significant effect on the disease. The clinical outcome was related to factors such as the age of onset, timing of treatment, and presence of tumors.

**Conclusion:** In conclusion, specific antibody tests should be performed as early as possible in suspected cases. Clinicians should actively administer immunotherapy and the management of the co-tumor. In addition, repeat antibody tests and image examinations following discharge from the hospital guide the maintenance protocol of immunotherapy.

## Introduction

Investigations in the past 10 years have revealed a new category of antibody-mediated neurological diseases against the cell surface and synaptic proteins. These diseases are characterized by autoantibodies (ABs) against neuronal proteins involved in synaptic signaling and plasticity (1). Based on different antigens, there are 16 known types of the disease, including CASPR2 (contact protein-related protein 2), GABA(A/B)R (type A/B receptor for gamma-aminobutyric acid), LGI1 (leucine-rich glioma inactivation protein 1), NMDAR (N-methyl-D-aspartate receptor), AMPAR (α-amino-3-hydroxy-5-methyl-4-isoxazole propionic acid receptor), and others (1). Of these, AMPAR-associated encephalitis was initially described in 2009. It was reported in 10 patients, all presenting with limbic encephalitis (LE), thus suggesting antigenic targeting of AMPAR GluA1 or GluA2 subunits by an immunologic detection of the cerebrospinal fluid (CSF) and serum antibodies (2). The present research finds that antibodies from patients with anti-AMPAR encephalitis selectively eliminate the surface and synaptic AMPARs, thereby resulting in a homeostatic decrease in the inhibitory synaptic transmission and increased intrinsic excitability, which in turn may contribute to memory deficits and epilepsy (3). Subsequently, researchers reported on AMPAR-Ab-related encephalitis cases and found that LE was the most common manifestation, sometimes misdiagnosed because of overt psychiatric symptoms or hyponatremia. The majority (64%) of patients have an underlying tumor. In addition, a significant proportion (32%) of patients have co-morbid paraneoplastic antibodies, suggesting concurrent autoimmune phenomena. While the disease responds significantly to immunotherapy, its long-term outcome is influenced by the presence of paraneoplastic antibodies and associated paraneoplastic symptoms or tumors (4). In total, 83 cases of AMPAR-E have been confirmed by immunoassay and reported publicly, of which only 55 cases have detailed information on the clinical characteristics (5). Herein, we present in the detail of six confirmed novel cases. We aimed to clarify the clinical course and prognosis of the disease in the light of previous reports.

## Materials and Methods

In the past 5 years, six cases of AMPAR-E were diagnosed successively at the Qilu Hospital, Shandong University, according to the diagnostic criteria of autoimmune encephalitis (AE) published in 2016 (6). The male-to-female ratio was 5:1, ranging in age from 2 to 70 years (average 42 years). The median time from symptom onset to definitive diagnosis was 5.4 weeks (range 1.3–17 weeks). Of these, only case 2 was pathologically diagnosed with type B2 thymic carcinoma, and this was 1 month before the disease onset. We reviewed the clinical characteristics of these six patients, including (1) clinical presentation: cognitive and psychiatric symptoms, systemic manifestations, and epilepsy; (2) laboratory tests, including routine serological tests, CSF cell counts, protein concentrations, and electrolytes (the serum and CSF samples were all sent to the Simcere Testing Center to assess auto-antibodies to the NMDAR, LGI1, AMPA1 and AMPA2, CASPR2, and the GABABR in the serum and CSF by Cytometric Bead Array); (3) imaging studies: to determine comorbid intracranial structural and functional lesions and to exclude systemic tumors; (4) electrophysiological monitoring; and (5) treatment and follow-up, including therapy, the degree of improvement, and ancillary examinations.

We obtained clinical information from the medical records of inpatients or outpatients. The follow-up was conducted by telephone or outpatient visit. This study standardized the assessment of patients using internationally recognized scales, such as the Modified Rankin Scale (mRS) and the Clinical Assessment of Autoimmune Encephalitis Scale (CASE). The relationship between the onset, treatment, and outcome was elucidated based on statistical results. Moreover, we explored the risk factors for poor prognosis. Office version 2019 was applied for the statistical analyses and graph production.

This study was conducted in accordance with the tenets of the Declaration of Helsinki and was approved by the ethics committee of the Qilu Hospital of Shandong University (NO. KYLL-202008-044). Written informed consent was obtained from all the participants and their legal guardians.

## Results

### Clinical Data

#### Clinical Presentation

**Figure 1:**
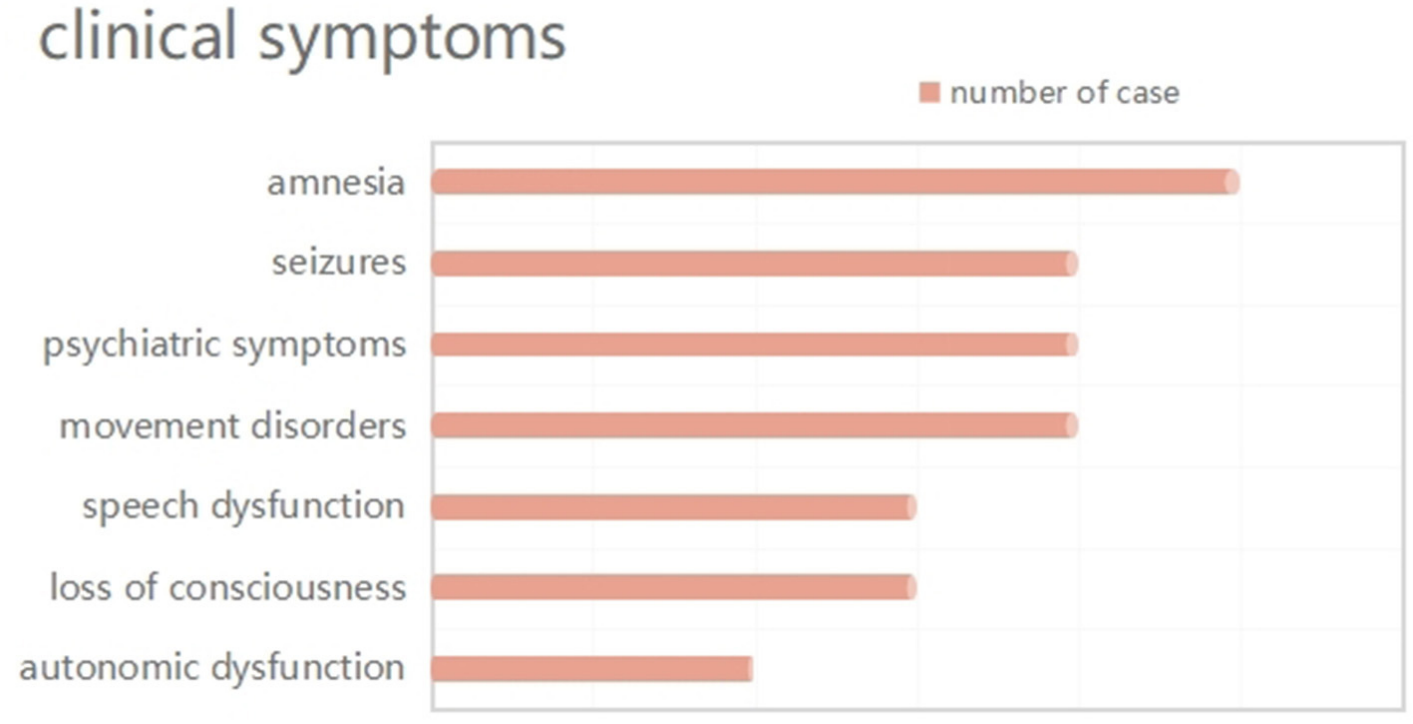
Distribution of clinical manifestations.

None of the six patients had obvious symptoms of viral encephalitis or other infections, prior to the disease onset. One patient had a mildly elevated temperature on admission. They primarily manifested as LE (Figure 1): amnesia, seizures, psychiatric symptoms, speech dysfunction, and loss of consciousness in five (83.33%, four cases as the initial symptom), four (66.67%, two cases as the initial symptom), four (66.67%), three (50.00%), and three (50.00%) cases, respectively. Four cases developed movement disorders (usually manifested as dystonia). Autonomic symptoms occurred in two cases, including incontinence and central hypoventilation. The symptoms were standardized and assessed in all patients with mRS and CASE scores ranging from 3 to 4 and 4 to 14 points, respectively.

#### Laboratory Findings

A total of six patients were positive for the surface-specific antibody test (AMPAR-Ab). In addition, the ratio of the two subtypes (GluA1:GluA2) was 2:5 (Table 1). Except for case 3 and 6, whose antibodies were detected only in the serum, the antibodies were positive both in the serum and CSF of the remaining patients. In addition to AMPAR-Ab, other types of neuronal ABs, such as acetylcholine receptor antibodies (AchR-Ab), NMDAR-Ab, and GABABR-Ab were detected in three cases. Two cases were positive for the combined anti-thyroid and/or systemic ABs. Interestingly, case 4 had both anti-AMPA type 1 and 2 and anti-NMDAR antibodies in the CSF, thus indicating the possibility of concurrent AE in both types and/or two subtypes of a similar type. All six patients were negative for paraneoplastic antibodies and oligoclonal electrophoresis. The CSF pressure was within normal range. In the routine test for the CSF, the white blood cell count, primarily lymphocyte was mildly high in three cases. Moreover, the protein concentration and immune index were also high (Table 1). The tumor marker test only found mildly increased CA724 levels in four cases. There were no obvious abnormalities in the serum electrolytes, and none of them were combined with hyponatremia.

**Table 1:** Clinical data.

**No**.	**Age (year)**	**Sex (M/F)**	**Initial symptom**	**Other symptoms**	**Cranial MRI**	**EEG**	CSF cell (/mm^**3**^) and protein (g/L)^*^	**Tumor**	**Treatment**
1	2	M	Seizure	Drowsiness	Normal	Spike-slow complex wave and slow wave activity	1; 0.19	–	Steroids + IVIg
2	26	M	Amnesia	Drowsiness, irritability, general seizure, dystonia, no speech, incontinence	Abnormal signals (bilateral hippocampal)	Normal	10; 1.18	Thymic carcinoma	Steroids + IVIg + CTX
3	62	M	Amnesia	Drowsiness, irritability, dystonia, speech disorder	Atrophy (bilateral hippocampal)	Slow wave activity	8; 0.69	–	Steroids + IVIg
4	25	M	Amnesia	Absence seizure, Involuntary movements, hyperhidrosis	Abnormal signals (the frontal and insular lobes)	Normal	2; 0.25	–	Steroids + IVIg
5	70	M	Seizure	Amnesia, balderdash coma, Speech disorder	Abnormal signals (right hippocampal)	slow wave activity	12; 0.45	–	Steroids + IVIg
6	65	F	Amnesia	Dysphoria, limb trembling	NORMAL	NORMAL	6; 0.33	–	steroids + IVIg

*EEG, electroencephalogram; MRI, magnetic resonance imaging*.

* *CSF: the normal number of cells is not >5 /mm^3^; the protein concentration generally ranges from 0.15 to 0.45 g/L*.

#### Imaging and Electrophysiological Examination

**Figure 2:**
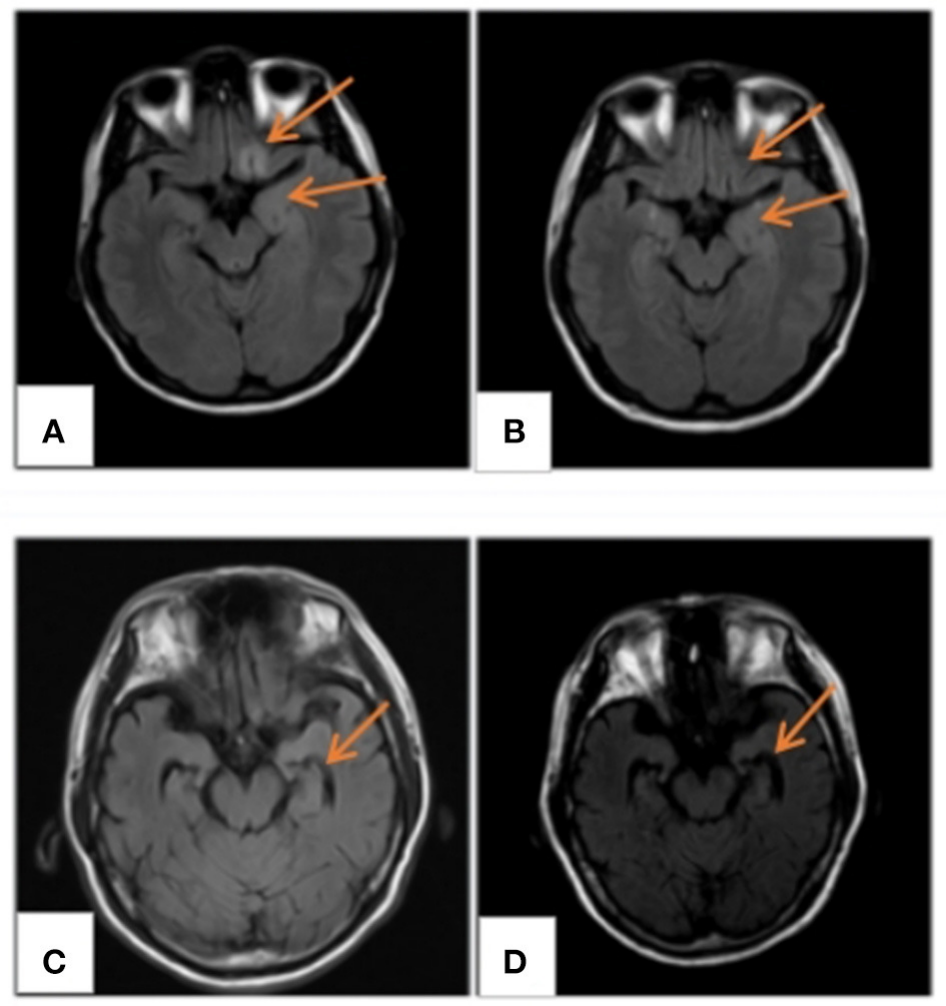
MRI findings. **(A)** MRI FLAIR/T2 of case 4 displaying abnormal signals in the frontal and insular lobes, **(B)** normal after 3 months; **(C)** hippocampal atrophy in case 3; and **(D)** atrophy development 4 months later. MRI, magnetic resonance imaging; FLAIR/T2, fluid-attenuated inversion recovery.

**Figure 3:**
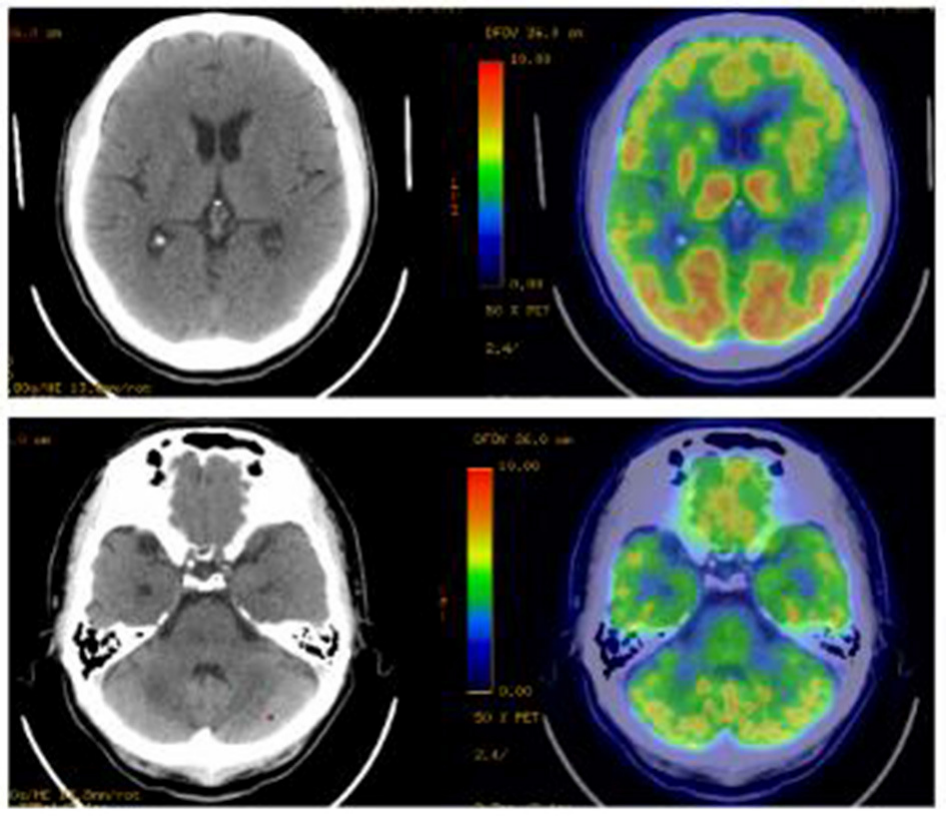
PET-CT imaging. Bilateral temporal lobe and cerebellar reduced fluorodeoxyglucose metabolism level. PET-CT, Positron Emission Tomography and Computed Tomography.

All patients underwent cranial magnetic resonance imaging (MRI). We observed increased fluid-attenuated inversion recovery (FLAIR)/T2 signal abnormalities in three cases, predominantly in the limbic lobe (Figure 2). While two cases had no positive findings, another case through several re-examinations revealed progressive bilateral hippocampal atrophy. Cranial positron emission computed tomography (PET-CT) revealed bilateral temporal lobe and cerebellar reduced fluorodeoxyglucose (FDG) metabolism level (Figure 3). We conducted a CT scan of the chest, abdomen, and pelvis in five case. Thymoma and pulmonary nodules were observed in cases 2 and 5 (unknown nature), respectively. All patients underwent a routine scalp electroencephalogram (EEG) examination. Only one patient caught epileptiform activity and three patients had slow wave activity in the course of the disease (Table 1).

### Treatment and Outcomes

All patients were treated with first-line immunotherapy following diagnosis, at an interval of 1.4 to 17 weeks (median duration, 5.4 weeks) from the symptom onset to the start of treatment. Six patients received both intravenous immunoglobulin (IVIG; 0.4 g/kg/d for 5 days) and steroids, namely intravenous methylprednisolone [500–1,000 mg (15–30 mg/kg/d) for 3–5 days], followed by oral prednisone (1 mg/kg/d) transition at the end of the course, supplemented by symptomatic support. Case 2 had three successive courses of hormonal shocks, which were generally effective. They relapsed after improvement, during which she was admitted to the ICU because of severe symptoms and central hypoventilation. Moreover, she underwent a second-line treatment plan [cyclophosphamide (CTX) 0.8 g qw, three times]. The improvement rate of CASE and mRS was 0.29–0.80 and 0–0.67, respectively, during discharge.

### Follow-Up

All patients were followed up by telephone or outpatient visits for 6 to 48 months with a mean period of 16 months. The overall prognosis of the six patients was good, with a 50–100% improvement in CASE (average 78%) (Table 2). Only case 3 recurred following discharge from the hospital, primarily manifesting frequent seizures. Following treatment with intravenous immune globulin, oral hormones, and the addition of immunosuppressants, the patient still experienced significant seizures, predominantly convulsions on the right side of the face and right hand. By the last follow-up (6 months), the patients were administered oral hormones (25 mg qd) and clonazepam combined with levetiracetam to treat epilepsy. The CASE scores improved by 60%. Cases 1 and 4 were in complete remission, 3 months following discharge, with a 100% improvement in CASE scores. Despite no relapse in case 2, the condition persisted and its symptoms improved slightly following radiation therapy for thymic carcinoma. However, its prognosis was relatively poorest, with an unchanged mRS score and an improvement rate of 50% in CASE. Five patients were rechecked for AE antibodies (Table 3). Of these patients, case 4 turned negative for AMPA1 and NMDAR antibodies and had a reduced AMPA2 titer. In the remaining patients, two cases turned negative, one case had a reduced titer, and another case had no significant change in titer. We rechecked two patients for cranial imaging following their discharge. Abnormal cortical signals in the acute phase disappeared in case 4. In contrast, hippocampal atrophy remained in case 3. We reviewed the EEG in two patients, with no further epileptic wave distribution found in case 1 and a persistent slowing of the background activity in case 3.

**Table 2:** Prognosis analysis.

**No**.	**Time interval (week)**		**On admission**		**On discharge**		**Final follow-up**		**Follow-up time (month)**
	**Onset to diagnosis**	**Onset to treatment**	**mRS**	**CASE**	**mRS**	CASE (%)^*^	**mRS**	CASE (%)^*^	
1	1.5	1.4	4	4	2	1 (75)	0	0 (100)	48
2	4	4.6	4	14	4	10 (29)	4	7 (50)	21
3	17	17	3	10	2	5 (50)	2	4 (60)	6
4	3	4	3	5	1	1 (80)	0	0 (100)	6
5	1.3	1.3	4	11	4	7 (36)	2	3 (73)	6
6	4	4	3	7	3	4 (43)	0	0 (100)	9
Mean	5.4	5.4	3.5	8.5	2.7	4.7 (41)	1.3	2.3 (80)	16

*CASE, Clinical Assessment of Autoimmune Encephalitis Scale*.

* *(%): percentage of CASE value reduction since admission*.

**Table 3:** Ab testing.

		**CASE1**	**CASE2**	**CASE3**	**CASE4**			**CASE6**
**Ab subtypes**		**AMPA1**	**AMPA2**	**AMPA2**	**AMPA1**	**AMPA2**	**NMDA**	**AMPA2**
initial titer	serum	1:32	1:32	1:32	1:32	1:32	1:10	1:32
	CSF	(–)	1:1	1:32	1:3.2	1:32	1:10	(–)
review titer	serum	(–)	1:32	1:10	(–)	1:100	(–)	(–)
	CSF				(–)	1:1	(–)	

*AB, autoantibody; AMPA, anti-α-amino-3-hydroxy-5-methyl-4-isoxazolepropionic acid; CASE, Clinical Assessment of Autoimmune Encephalitis Scale*.

## Discussion

AE represents a heterogeneous group of autoimmune disorders in the CNS, characterized by ABs to neuronal targets localized at central synapses, such as ionotropic or G-protein-coupled receptors, presynaptic proteins, or ion channels (1). In autoimmune encephalitides, the antibodies bind to extracellular epitopes of cell-surface proteins and cause reversible neuronal dysfunction (1). These features may explain better outcomes in patients with autoimmune encephalitides, compared to those in patients with antibody-mediated neurologic syndromes against intracellular proteins (7). The annual incidence of all types of encephalitis is about 5–8 cases/100,000, of which 40–50% are uncertain (8). A prospective, multicenter, population-based study demonstrated that autoimmune disease occurs following an infection, and is usually the third most common cause of encephalitis following viral and acute disseminated encephalomyelitis (8).

AMPAR is an autoantibody target in autoimmune encephalitis patients. The initial description of the encephalitis associated with these antibodies was published in 2009 and included 10 patients, all with LE. They had CSF and serum antibodies that reacted with the neuropil of rat brain and the cell surface of rat hippocampal neuron culture, thus precipitating and characterizing the target antigens as the GluA1 or GluA2 subunits of the AMPAR. This in turn supported the antibody-mediated pathogenesis that the patient's antibody alters synaptic localization and the number of AMPAR (2). AMPAR is an ionic glutamate receptor that mediates most rapid excitatory transmission in the brain, and it is an important mechanism for synaptic transmission regulation. Moreover, it is important for synaptic plasticity, memory, and learning (9). The majority of AMPA receptors are tetramers composed of GluA1, 2, 3, or 4 subunits that combine in a brain region-dependent manner (10). The highest levels of GluA1/2 and GluA2/3 receptors are found in the synaptic CA3-CA1 region of the hippocampus, subiculum, cerebellum, caudate-putamen, and cerebral cortex (11). Antibodies against extracellular epitopes of GluA1 and GluA2 have disease relevance in causing AE with prominent limbic dysfunction. In contrast, antibodies against GluA3 do not appear disease relevant and are not detected in most patients with Rasmussen's encephalitis or other syndromes (1). The effects of patient antibodies on cultures of live rat hippocampal neurons were determined with immunostaining, Western blot, and electrophysiological analysis, thereby establishing that antibodies from patients with anti-AMPAR encephalitis selectively eliminate the surface and synaptic AMPARs. This eventually results in a homeostatic decrease in inhibitory synaptic transmission and increased intrinsic excitability, which may contribute to memory deficits and epilepsy that are prominent in such patients (3).

According to previous reports, the age of onset of AMPAR-E is heterogeneous (ranging from 14 to 92 years, mean 53.1 years), with a female preponderance of 36/55. The patients included in this study had a male to female ratio of 5:1, and their age ranged from 2 to 70 years with a mean age of 42 years. Taken together, the disease has no significant gender distribution and is most common in middle-aged adults. However, it affects all age groups, ranging from children to the elderly. The median time from symptom onset to the diagnosis of AE was 5.4 weeks (range, 1.3–17 weeks). Except for case 2, who was diagnosed with malignant thymoma because of ptosis 1 month before the disease onset, none of the cases had obvious prodromal symptoms. Considering the development of other neurological immune diseases, the development of AMPAR-E may involve changes in the tolerance-inducing mechanism in the thymus. There was significant heterogeneity in the clinical presentation and the severity at onset, with mRS and CASE scores of 3–4 and 4–14, respectively. All cases initially manifested limbic encephalitis, with five cases demonstrating memory loss. A child manifested only generalized seizure and impairment of consciousness with a CASE score of 4. Considering the diagnosis was made shortly after its onset (11 days), the severity of symptoms may be related to the duration of the disease and the onset age. Case 2 demonstrated a combination of malignancy and poor general condition (CASE score = 14 points). We performed a neuronal antibody profile, including anti-intracellular, extracellular, and synaptic vesicle antigens on the serum and CSF, which generated positive anti-acetylcholine receptor antibodies (AchR IgG), in addition to AMPAR-Ab. Therefore, the presence of malignancy was associated with more severe systemic symptoms.

We observed an abnormal signal of craniocerebral T2 FLAIR in three cases during the MRI examination, thus indicating the diagnosis of marginal LE (12). A negative MRI did not rule out the disease. For example, cases 1, 3, and 6 manifested no typical LE changes, which may be related to their age and disease duration. The EEG may reveal no obvious abnormalities other than epileptic waves and inflammatory slow waves. In addition, the routine CSF examination may demonstrate a slight increase in the white blood cell count and protein, with no obvious specific changes. This necessitates an early detection of antibodies in suspected cases.

Most (5/6 in this group) patients had significant improvement in their neurological symptoms and in the degree of CASE improvement (29–80%, average 52%) following first-line immunotherapy, consistent with the AMPAR-AE case series and other frequent AE subtypes (13) (Table 2). Case 2 was admitted to the ICU because of severe central hypoventilation after three rounds of immunotherapy, in addition to second-line drugs. However, we did not observe any significant effect. The neurological symptoms improved slightly during hospitalization and recurred again. Case 3 also benefited from the addition of second-line drugs because of post-discharge fluctuations in symptoms. Therefore, second-line therapy should be initiated immediately in patients with AE who have severe clinical symptoms and poor response to first-line therapy to maximize the improvement.

**Figure 4:**
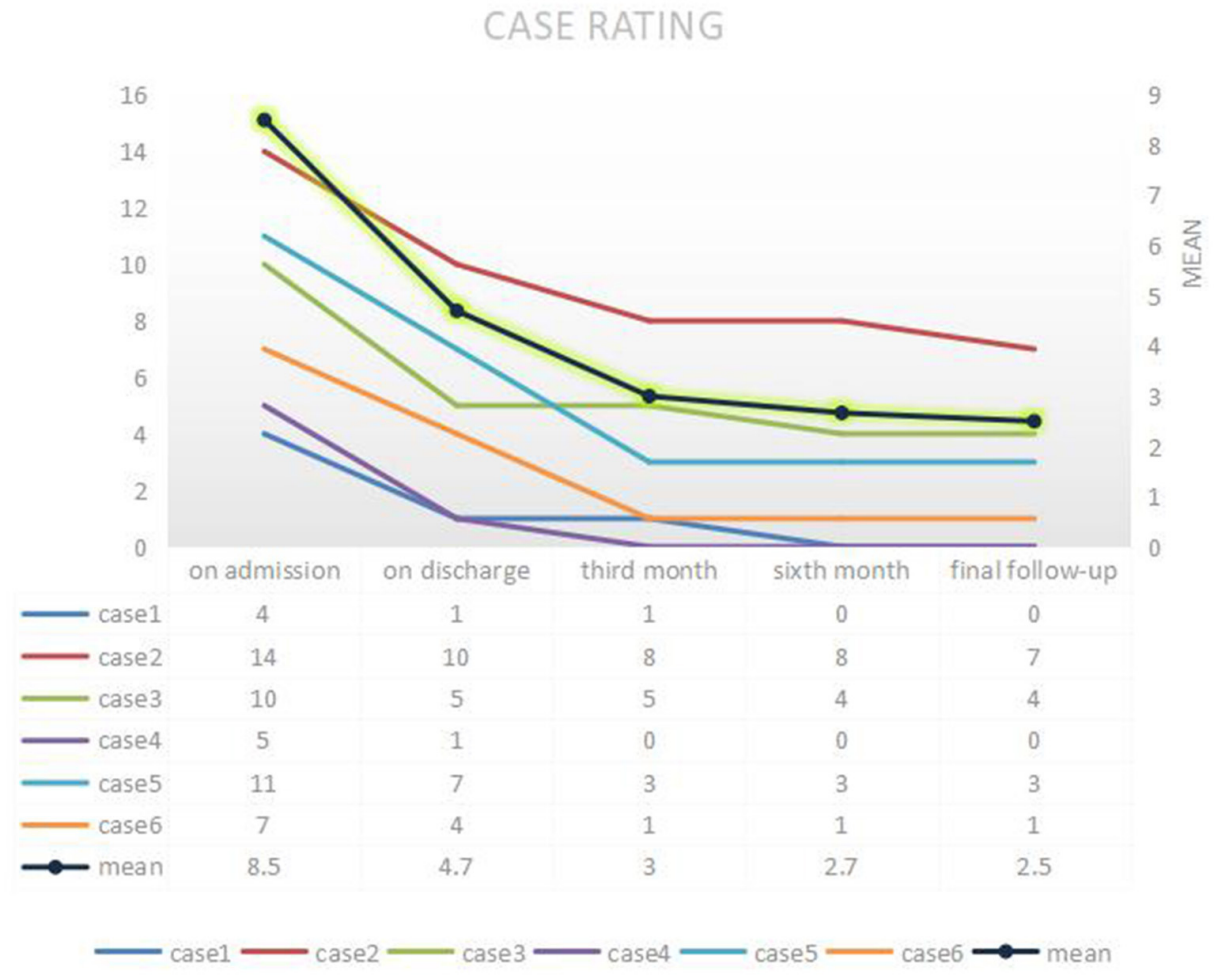
Follow-up of CASE. CASE, Clinical Assessment of Autoimmune Encephalitis Scale.

A study conducted in 2015 suggested that patients with AMPAR antibodies show less substantial recoveries than those with other types of autoimmune encephalitis [NMDAR, LGI1, or GABA(B)R] (4). Poor prognosis may be related to factors, such as co-tumor, the age of onset, and delayed treatment. Most patients recovered well during a follow-up period of 6 to 48 months, with 50-100% (average 78%) improvement in the CASE scores at the last follow-up. Figure 4 depicts the CASE regression of all patients. Of these patients, four cases were treated at an early stage of the disease (≤4 weeks) with first-line treatment. Furthermore, the improvement rate of the last follow-up was >70%, in which two cases comprised young patients without any residual symptoms. However, an unclear diagnosis for a prolonged period delayed case 3. Moreover, its prognosis was general, thus suggesting all suspected cases should be immediately tested for antibodies to initiate rapid treatment for maximizing the recovery. Older age and more severe initial symptoms may also indicate a poor prognosis. The patient with co-tumors (case 2) had the lowest rate of improvement and still suffered from mild psychiatric symptoms at the last follow-up (21 months). The logical analysis of the AMPARE outcomes initially demonstrated that the presence of psychiatric symptoms predicted the presence of tumors (*z* = 2.06, *p* = 0.040, or 4.9 [95% CI: 1.2–25.3]) (5). Researchers have established an association between mental symptoms and disease-related malignancies and poor prognosis. The patient was discharged from the hospital with obvious symptoms (CASE score = 10 points). However, there are instances of recurrences even after the first- and second-line treatment. Therefore, the tumor should be carefully reviewed in patients with concomitant significant psychiatric abnormalities. Interestingly, the patient did not develop any fluctuation of encephalitis symptoms in the 6 months following discharge after radiation treatment. Nonetheless, his symptoms did not improve after 1 year, thereby suggesting the tumor should be treated immediately as a predictor of poor prognosis.

The determination of antibody titer is of great significance in the diagnosis and evaluation of prognosis. AMPAR AB detection was considered specific for AMPAR encephalitis in patients suspected of autoimmune encephalitis, with a low serum positive rate in people with neurological disease and their healthy counterparts (<0.1%) (14). Despite no definite correlation between the initial titer and the severity of the disease, changes in antibody titers over the course of the disease may be instructive. In 2010, Bataller et al. (15) found that a decrease in AMPA antibody titers restored the patient's ability to form new memories. Six patients included in this study had initial antibody titers of +~++, which did not correlate significantly with the CASE scores at the disease onset. Five patients underwent at least onc anti-AE antibody test following their discharge. Except for case 2, which had a relatively poor prognosis, the antibody titers of blood/CSF of the remaining three patients were significantly reduced at the last follow-up. This in turn was consistent with an improvement of their clinical symptoms. Despite being negative for AMPA1 and NMDAR antibodies, 6 months following discharge, case 4 was still positive for AMPA2 antibodies. Her clinical symptoms had almost disappeared (last follow-up CASE score = 0). Nonetheless, she should still be maintained on low-dose immunotherapy to avoid relapse, thus suggesting the results of specific antibodies may be a more accurate assessment of disease progression than the clinical symptoms. Moreover, data from the AE case series in 2014 suggested that patients with good outcomes saw a faster and greater decrease in CSF antibodies than those with poor outcomes (16). Therefore, periodic antibody titers may be significant in guiding the maintenance of patients and the timing of the discontinuation of therapy.

Imaging examination and EEG are indicative for disease diagnosis and referral. The positive rates of cranial imaging and EEG were 75 and 50%, respectively, thus suggesting combined limbic encephalitis. The MRI of case 3 was relatively atypical, with no obvious positive findings in the early stage of the disease, followed by hippocampal atrophy 1 year later. This eventually suggested possible progression of the disease, consistent with a relapse in its clinical course. Notably, PET/CT displayed reduced metabolic signals in the temporal lobes and cerebellum. A recent study found that brain 18F-FDG PET/CT (91.3%) is more sensitive than MRI in the acute phase of AE, primarily revealing hypometabolism of the brain lobes. Moreover, this molecular imaging technique may be better suited as an early biomarker of the disease (17). However, it has not been widely used in this disease owing to the high cost and radiation intensity of the test. EEG/MRI of cases 1 and 4 were negative at the last follow-up. However, case 3 still had hippocampal atrophy (MRI) and background hypoactivity (EEG), consistent with the clinical outcomes of the disease. Therefore, routine imaging and EEG in patients with AE exert a positive effect on the diagnosis and an assessment of the disease course.

## Conclusion

In conclusion, AE can be diagnosed across all age groups, mostly in young adults. It often presents with LE encephalitis syndrome and partly with tumor. The disease responds favorably to immunotherapy. Furthermore, second-line therapy should be initiated immediately in patients with fluctuating disease following the first-line therapy. The long-term prognosis depends on the age of onset, the timing of treatment, the presence of the tumor, and other factors. Repeated detection of antibody titers and imaging review can provide instructions for therapeutic maintenance.

Our study presented in detail six confirmed AMPAR-AE cases that have not yet been reported and analyzed the clinical and prognosis of the disease in the light of previous reports. The value of AE-AB repeated testing for evaluating the progress and guiding the treatment was initially emphasized. However, the small sample size substantially limits the power for data analysis. More cases should be included for retrospective or prospective cohort studies to clarify the adverse prognostic factors of AMPAR-AE and achieve better disease outcomes.

## Data Availability Statement

The original contributions presented in the study are included in the article/supplementary material, further inquiries can be directed to the corresponding author/s.

## Ethics Statement

The studies involving human participants were reviewed and approved by the Ethics Committee of Qilu Hospital of Shandong University (No. KYLL-202008-044). Written informed consent to participate in this study was provided by the participants' legal guardian/next of kin. Written informed consent was obtained from the individual(s), and minor(s)' legal guardian/next of kin, for the publication of any potentially identifiable images or data included in this article.

## Author Contributions

XL contributed to the conception of the study. KW and YS contributed significantly to analysis and manuscript preparation. QD collected the clinical data. R-rZ performed the follow-up. KW performed the data analyses and wrote the manuscript. HW and SQ helped perform the analysis with constructive discussions. All authors contributed to the article and approved the submitted version.

## Conflict of Interest

The authors declare that the research was conducted in the absence of any commercial or financial relationships that could be construed as a potential conflict of interest.
